# Nonintrusive Dynamic Pressure Monitoring of Sloshing Liquid Using a Hierarchically Microstructured Flexible Sensor

**DOI:** 10.1021/acs.langmuir.6c01078

**Published:** 2026-06-18

**Authors:** Parul Thapa, Shrutidhara Sarma

**Affiliations:** Department of Mechanical Engineering, IIT Jodhpur, Jodhpur 342030, Rajasthan, India

## Abstract

Monitoring dynamic pressure in sloshing liquids is critical for the safety of storage, transportation, and aerospace systems, as uncontrolled sloshing can increase structural loads and cause vehicle instability or containment failure. Conventionally, such measurements are done using rigid pressure transducers that are flush-mounted through drilled ports. This process disturbs the flow, limits the repositioning of sensors, and is unsuitable for curved or flexible tank surfaces. In this study, a hierarchically microstructured flexible pressure sensor (FlexiHMS) was employed to monitor slosh-induced wall pressures and benchmarked against a commercial piezoelectric (PZT) sensor. Controlled sloshing experiments were conducted in a lab-developed rectangular tank at 22%, 30%, and 70% fill volumes under base excitation from 0.8 to 1.2 times the first-mode natural frequency. Free-decay tests validated the test protocol, where dominant frequencies deviated from theoretical values by less than 5%. FlexiHMS successfully captured peak dynamic pressures ranging over (0.3–127) Pa and closely followed the expected resonant amplification behavior. Frequency-domain analysis using fast Fourier transforms and steady-state boxplots revealed pronounced nonlinear pressure characteristics near resonance, with increased dispersion and intermittency. While both sensors demonstrated repeatable cycle-to-cycle responses across multiple trials, FlexiHMS continued to exhibit a measurable pressure response under conditions where the PZT response diminished, especially at higher fill volumes. Our results highlight the potential of flexible pressure sensors like FlexiHMS for conformal, nonintrusive, dynamic pressure monitoring in fluid-structure interaction systems where conventional rigid sensor mounting is impractical.

## Graphical abstract

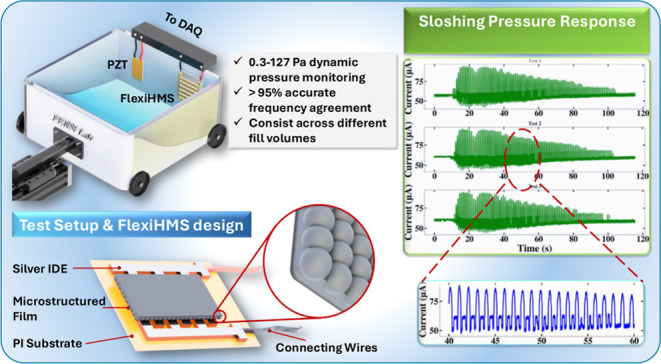

## 1 Introduction

Sloshing of liquids in partially filled containers generates fluctuating and transient pressures on the container walls that can pose serious safety risks. These fluctuations arise from external disturbances such as turbulence, acceleration, or vehicle maneuvering during transportation^1^ and can lead to structural damage and vehicle destabilization, especially when excitation frequencies approach the natural sloshing modes of the liquid. Reliable measurement of such dynamic pressures is therefore essential for understanding fluid-structure interactions and ensuring operational safety of liquid-carrying systems.

Conventional approaches for measuring sloshing-induced pressures usually employ rigid, high-frequency dynamic pressure transducers, such as piezoelectric or piezoresistive sensors.^2,3^ These sensors typically require flush mounting through drilled holes in the container wall and precise sealing to ensure accurate measurements. For example, Pistani and Thiagarajan used Kulite XCL series transducers to study sloshing in liquefied natural gas tanks used in marine vessels.^4^ Similarly, Zhao et al. used six different types of rigid sensors, such as piezoelectric accelerometers, piezoresistive and capacitive pressure sensors, laser displacement sensors, and liquid level sensors, to examine the effect of free-standing structures on liquid sloshing in a water tank.^5^ More recently, Jentzsch et al. demonstrated that nonflush mounting of rigid pressure sensors significantly reduced the frequency response range by introducing frequency-dependent attenuation and phase lag in measurements.^6^ Lugni et al. used rigid piezoelectric pressure sensors to investigate localized wave impact loads and highlighted the sensitivity of measured peak pressures to sensor placement and structural interaction effects.^7^ Although rigid pressure sensors provide a high-frequency response, their rigid form factor, reduced performance while not flush-mounted, and strong sensitivity to installation position limit their scalability and practical adaptability. Moreover, intrusive installation requirements, structural disturbance effects, and incompatibility with curved or thin-walled structures further restrict their suitability for emerging fluid structure interaction systems.

Flexible pressure sensors can be directly adhered to surfaces without invasive modifications, and offer a simple, lightweight, and conformal alternative for distributed pressure monitoring. They are compatible with hierarchically microstructured designs that create extra voids and sharp contact edges, which increases compressibility and significantly improve sensitivity, particularly in low-pressure regimes.^8^ For these advantages, flexible piezoresistive sensors have been widely explored in applications such as wearable devices, electronic skin, human–machine interfaces, and health monitoring of engineering structures.^9–16^ However, most existing studies evaluate these sensors primarily under static or quasi-static loading conditions. For example, Ma et al. tested carbon nanotube and polydimethylsiloxane (CNT/PDMS) micropyramid-based pressure sensors under quasi-static compression in low (>10 kPa) and medium (10–100 kPa) pressure ranges and demonstrated functionalities such as frequency-modulated spike response and spatial pressure mapping.^17^ Similarly, Zhu et al. characterized a polyurethane/silver nanowires (PU/AgNW) mesodome-based sensor using static compression calibration across low to medium pressure regimes (0–204.7 kPa) and demonstrated durability over 10^3^–10^4^ cycles.^18^ Cao et al. studied a porous polyurethane/reduced graphene oxide (PU/rGO) sensor under quasi-static compression (0–3.2 kPa) with low-frequency cyclic testing (0.1 to 0.2 Hz).^19^ Likewise, Oh et al. characterized CNT/PDMS micropyramid sensors through controlled benchtop compression tests up to 100 kPa for applications in robotic and wearable systems.^20^ While these studies demonstrated excellent material performance, they focused primarily on material design or microstructural optimization and remained confined to controlled static or quasi-static characterization, which did not adequately represent complex loading conditions encountered in real-world applications. In dynamic environments such as fluid-structure interaction systems, sensors experience continuous oscillations, transient pressure peaks and nonlinear fluctuations. Sensor performance under such conditions is influenced by hysteresis, response and recovery times, stability under cyclic loading, and can often lead to signal drift and reduced repeatability. Although flexible sensors have been widely applied in low-frequency applications such as physiological monitoring, their validation under higher-frequency conditions like sloshing is limited. In sloshing, pressure fields are inherently transient, spatially nonuniform, and are governed by resonance-driven amplification, wave breaking, and intermittent wall impacts.^5,7,21^ Usually, such behaviors are investigated using rigid MEMS-based microcantilever sensors or traditional pressure sensors. One of the recent works have employed stretchable sensors on flexible tank to monitor structural deformation and infer force at high strain locations, rather than to measure localized slosh-induced wall pressures.^22^ They also did not investigate resonance-dependent pressure response, fill level effects, or transient and steady sloshing behavior.

To address these gaps, the present study evaluated the performance of a previously developed hierarchically microstructured flexible pressure sensor (FlexiHMS) under controlled sloshing conditions, which provided a representative dynamic environment involving both periodic excitation and nonlinear fluid motion. Sloshing induced wall pressures were monitored in a laboratory-scale tank integrated with a linear motorized stage to generate controlled motions. Experiments were conducted at multiple liquid fill volumes and excitation frequencies spanning subresonant, resonant, and super-resonant regimes. A rigid pressure sensor was not used as a benchmark due to its intrusive mounting requirements and incompatibility with the intended conformal sensing approach. Instead, a commercially available flexible PZT sensor was used as a qualitative reference to compare response trends with FlexiHMS. Due to differences in sensing mechanisms, the comparison focused on relative response behavior rather than absolute pressure values. By combining time and frequency-domain analyses with statistical representations of steady-state peak pressures, this work demonstrates the feasibility of flexible sensors for dynamic pressure monitoring in sloshing environments and highlights their advantages for nonintrusive fluid-structure interaction studies. This study addresses the following key questions.

i. Is our flexible sensor (FlexiHMS) able to reliably capture dynamic, sloshing-induced wall pressures under continuously oscillatory sloshing?
ii. How do liquid fill volumes and excitation frequencies affect the magnitude, variability, and frequency content of the sloshing-induced pressure fluctuations?
iii. How does the dynamic response of FlexiHMS compare to that of the commercial PZT sensor in terms of repeatability and consistency?

## 2 Materials and Methods

### 2.1 Sensor Used

The experiment employed our previously reported FlexiHMS sensor, fabricated via a rapid and scalable laser engraving technique.^8^ Circular microstructures were patterned on an ABS substrate using a DPSS laser and subsequently transferred to a PDMS film via replica molding. The patterned PDMS film was made conductive by spray coating CNTs, which was later assembled with a hand-painted Ag interdigitated electrode (IDE) on a polyimide (PI) substrate. The entire sensing assembly was encapsulated within a thin PDMS layer to enhance mechanical robustness and environmental protection. The laser engraving technique used played an important role in generating irregular microstructures on the sensing film leading to the presence of sharp contact points and voids. These features improved the sensor’s deformability as well as sensitivity by enabling progressive contact formation under compression. When a constant bias voltage of 1 V was applied, pressure-induced deformation of the microstructures increased the effective contact area between the CNT-coated sensing layer and the IDE that reflected a measurable increase in output current. For investigating the response of our FlexiHMS, a PZT sensor (28 μm PVDF film, LDT0–028 K, TE Connectivity) was used as a reference sensor. The key specifications of both sensors are listed in Table 1.

**Table 1:** Comparison of Key Specifications of FlexiHMS and PZT Sensors

sensors	sensitivity	response & recovery time	working freq range	working pressure range	other parameters
FlexiHMS (piezo-resistive)	25.33 kPa^–1^	360 ms, 390 ms	-	0–60 kPa	*R* ^2^ ≈ 0.98durability 1000 cycles (±4%)
	8.17 kPa^–1^	-do-	-	60–200 kPa	-do-
PZT (piezo-electric)	50 mV/g	-	0–90 Hz	-	operating temp. (0–85 °C) & storage temp. (−40–85 °C)

A preliminarily liquid oscillation test was conducted to evaluate the FlexiHMS response under liquid loading conditions. The sensor was attached at the bottom of a cylindrical tank filled with water and plunger with circular disc was subjected to sinusoidal excitation to move up and down using a motorized linear stage at a frequency of 1.5 Hz with displacement amplitudes of 2, 3, and 4 mm. The phase lag and amplitude linearity of the FlexiHMS was determined to be 31–49° and *R* ^2^ = 0.9786. The detailed waveform analysis and results are provided in Supplementary Appendix I. Preliminary thermal test was also conducted, and it showed high linearity under different temperatures (refer Appendix II, Supplementary).

### 2.2 Experimental Setup and Data Acquisition

For the sloshing test, a rectangular polypropylene (PP) tank with dimensions of 35 cm (length) × 24 cm (width) × 20 cm (height) was used, with water as the working fluid. The sensors FlexiHMS and PZT were affixed to one of the lateral walls of the tank using a thin layer of clear adhesive (Loctite Clear Silicone Waterproof Sealant, SKU/IDH 1700009). Figure 1a–c present the front, side, and top views of the tank along with the locations of FlexiHMS and PZT sensors, respectively. Both sensors were installed at a height of 45 mm above the tank bottom. Horizontally, FlexiHMS was placed 50 mm from the right wall, and PZT was placed 50 mm from the left wall.

**Figure 1:**
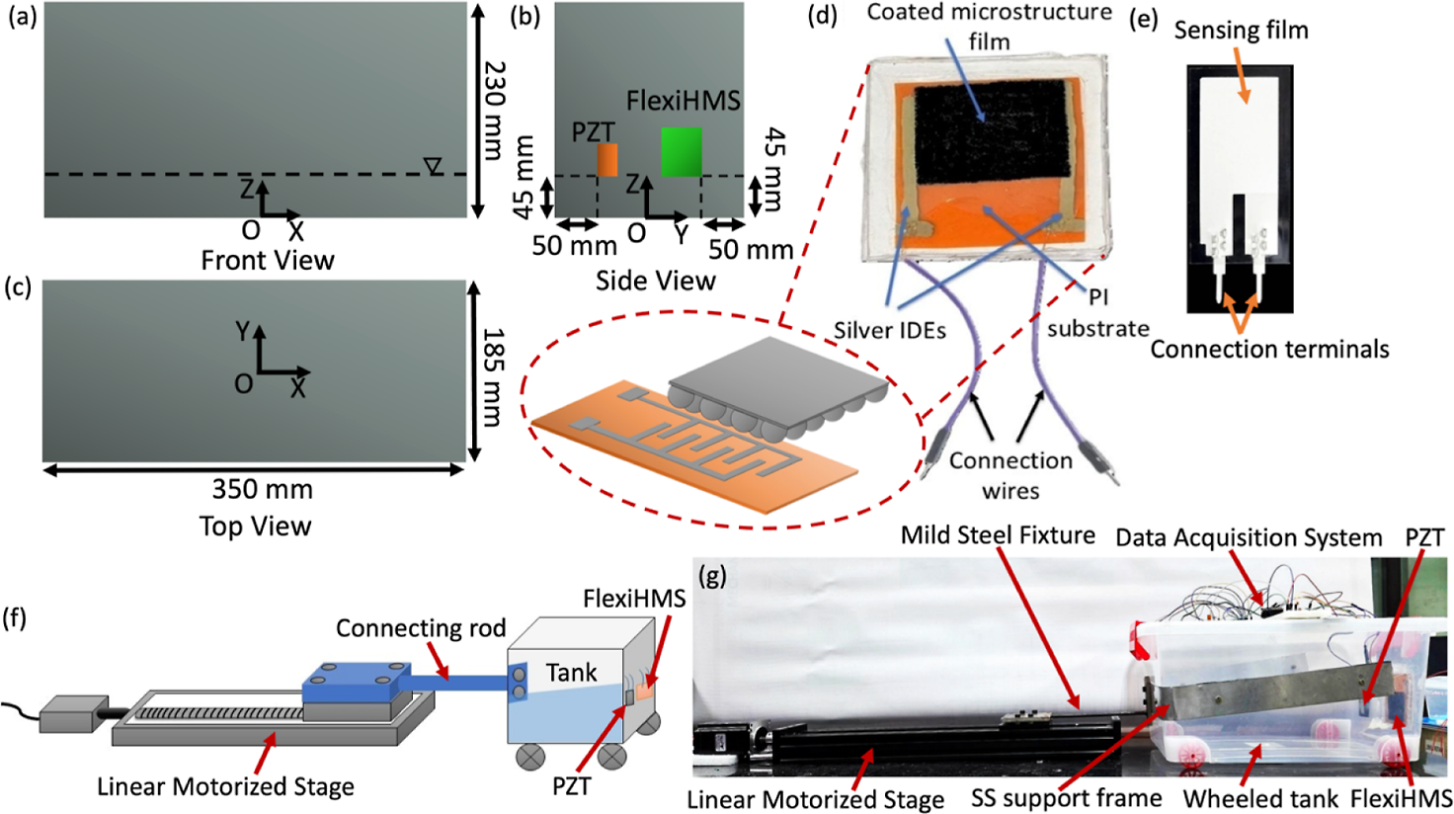
Sloshing tank configuration, sensor integration, and experimental setup for dynamic pressure sloshing. (a) Front, (b) side, and (c) top views of the rectangular tank with dimensions, and mounting locations of FlexiHMS and PZT sensors, (d) FlexiHMS sensor with a microstructured sensing film with an active sensing area of 40 × 40 mm^2^, (e) commercial PZT sensor with a sensing area of 10 × 20 mm^2^, (f) schematic of experimental setup showing a wheeled tank excited via linear motorized stage to generate controlled sloshing, (g) photograph of the actual experimental setup with a motorized stage connected to the tank along with sensors mounted.

Figure 1d,e show FlexiHMS and PZT sensors, respectively. The FlexiHMS output was recorded as the current variation corresponding to pressure fluctuations. The sensing electrodes of FlexiHMS were connected to a 24 bit ADC with an ESP32 microcontroller with an excitation voltage of 1 V via a Keithley sourcemeter. The output for FlexiHMS was recorded in terms of current, as it was more reliable.^8^ Side by side, the PZT sensor output was recorded in voltage by using a similar data acquisition setup. The outputs of both sensors were sampled at 1 kHz.

Figure 1f,g show the schematic and actual image of the experimental setup, respectively. A custom-made mild steel (MS) fixture was used to attach the tank rigidly to a linear motorized stage to ensure a secure and stable motion transfer during excitation. The fixture consisted of three welded MS components: a horizontal plate of size 75 × 75 × 6 mm bolted to the linear stage carriage, a vertical plate of size 15 × 75 × 6 mm fastened to the tank wall, and a 150 mm long, 6 mm diameter connecting rod between them. The fixture acted as a rigid mechanical link between the moving carriage of the linear stage and the tank assembly. The fixture was fastened to the tank frame using bolts and stainless steel (SS) support plate. The SS support plate was used to provide additional structural reinforcement and to distribute the transmitted motion uniformly across the tank wall to avoid localized stress concentrations at the bolted tank joints. The sloshing was generated using the linear motorized stage programmed via Python and supplied to the motor driver, with both speed and acceleration set to 150 000 units, to deliver controlled surge motion with excitation frequencies at different liquid fill volumes.

### 2.3 Test Procedure

Systematic experimental investigations were conducted under different liquid fill volumes (%) and excitation frequencies. The excitation range was selected based on the first-mode theoretical natural sloshing frequency (*f*_1_) of the tank for assessing both resonant and off-resonant conditions (0.8*f*_1_to 1.2*f*_1_). For a 2D rectangular tank, the sloshing frequency is given by^23^

1 $$f_{n}=\frac{1}{2\pi }\sqrt{g\frac{n\pi }{L}\tanh\left(\frac{n\pi }{L}h\right)}$$

where *f*_*n*_ denotes the nth-mode natural frequency, *h* denotes the liquid height, *L* is the tank length, and *g* is the gravitational acceleration.

For the present study, the tank was filled with water to 22%, 30%, and 70% of its height, and the corresponding first-mode frequencies were calculated using eq 1. The tank was excited using a linear motorized stage that generated periodic back-and-forth lateral motion. Unlike conventional harmonic excitation, the stage motion was acceleration-limited, and therefore the excitation frequency was not set directly, but depended on the programmed travel distance and acceleration. As the stage motion was nonsinusoidal, the effective sloshing frequency (*f*_*e*_) was determined experimentally from high-speed (60 fps) video recordings by counting wave-wall impacts as

2 $$f_{e}=\frac{N}{t}$$

where *N* is the number of impacts and *t* is the total time interval. The uncertainty resulting from the used method was below ±0.06% across all fill volumes (refer to Supplementary, Appendix III).

To validate the sloshing setup, the first-mode natural frequencies (*f*_1_) obtained using eq 1 were compared to the experimentally measured free-decay frequencies. A small manual disturbance was applied to initiate free oscillation, which was allowed to undergo free decay. The pressure signals were analyzed using fast Fourier transform (FFT) (FFT), as shown in Figure 2. For all fill volumes, the dominant frequencies obtained from both FlexiHMS and PZT closely matched the theoretical predictions (Table 2). For 22% fill volume, FlexiHMS exhibited a clear dominant peak at 0.946 Hz (Figure 2a) and PZT at 0.932 Hz (Figure 2b). For 30% fill volume, both sensors exhibited a dominant peak at 1.034 Hz (Figure 2c,d), and for 70% fill volume, FlexiHMS and PZT showed dominant peaks at 1.292 and 1.308 Hz, respectively (Figure 2e,f). This increase in dominant frequency with fill volumes was consistent with theoretical sloshing behavior, where the liquid depth results in an increase in the natural frequency of the tank. The small deviations between the theoretical and experimental frequencies (<5%) confirmed that the setup accurately reproduced the expected dynamic behavior of the liquid system and was suitable for subsequent sloshing experiments. This close agreement also suggested that mounting related compliance effects such as the influence of the adhesive layer were minimal.

**Figure 2:**
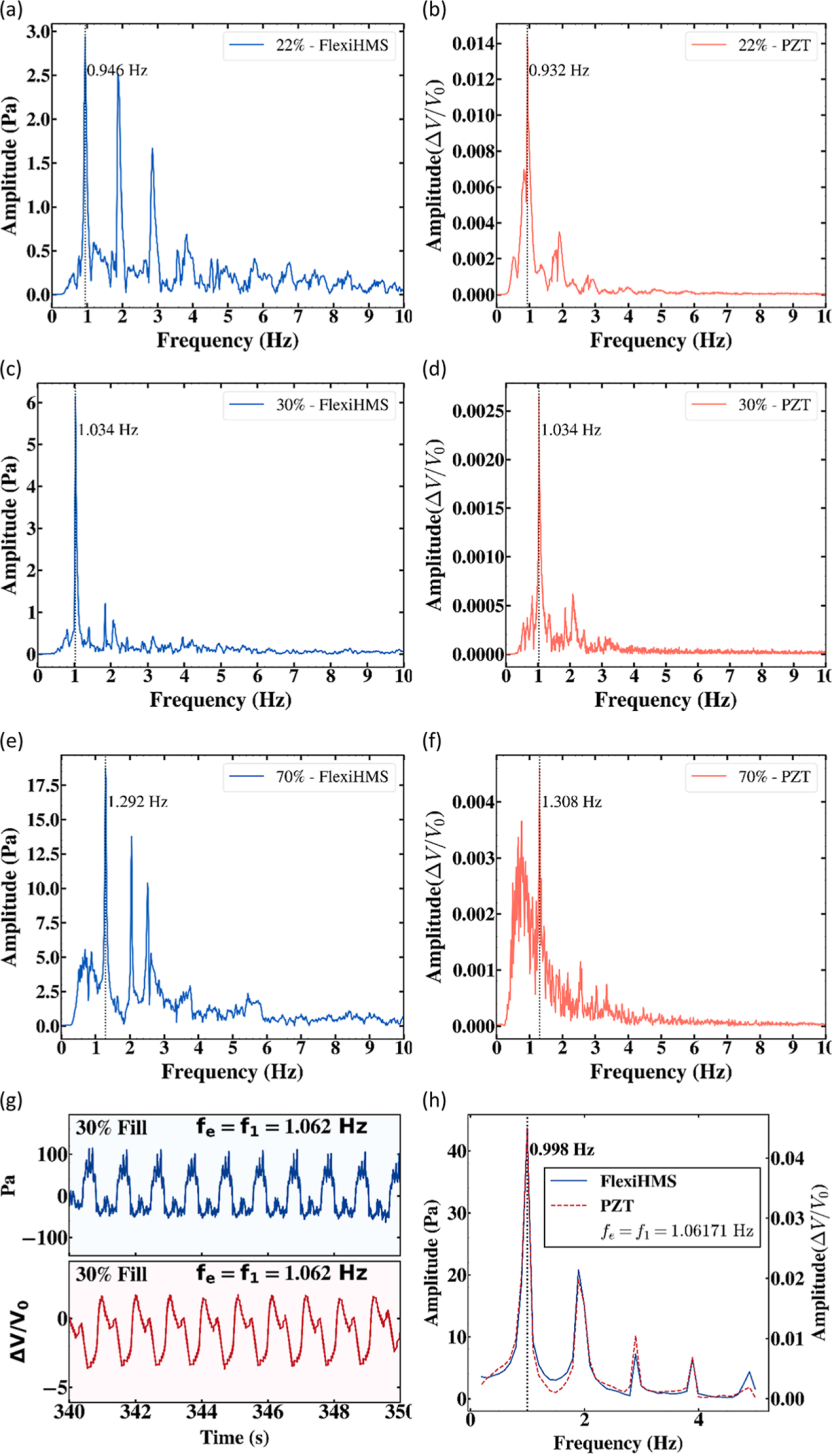
System validation: FFT spectra of free-decay at 22% fill volume measured using (a) FlexiHMS and (b) PZT; FFT at 30% fill volume obtained using (c) FlexiHMS and (d) PZT; FFT at 70% fill volume obtained with (e) FlexiHMS and (f) PZT. (g) Sample steady-state time domain plot at 30% fill volume under forced excitation (*f*_*e*_ = *f*_1_ = 1.06171 Hz) to demonstrate stable periodic oscillations and agreement between FlexiHMS and PZT; (h) corresponding FFT response of both sensors at 30% fill volume for *f*_*e*_ = *f*_1_ = 1.06171 Hz.

**Table 2:** Theoretical and Experimental First-Mode Resonant Frequencies at Each Fill Volume

fill volume (%)	liquid fill depth (cm)	theoretical 1st mode natural frequency *f*_*1*_ (Hz)	experimentally calculated *f*_*1*_ (Hz)		error b/n theoretical and experimental *f*_*1*_ (%)	
			FlexiHMS	PZT	FlexiHMS	PZT
22	4.6	0.9338	0.946	0.932	–1.31	0.19
30	6.2	1.0617	1.034	1.034	2.61	2.61
70	12.8	1.35	1.292	1.308	4.3	3.1

Since there was no calibration relation provided by the manufacturer to convert the voltage output to pressure readings for the commercial PZT sensor, the absolute amplitudes of the two sensors were not compared directly. Instead, the output distribution, trends in the frequency response, and repeatability characteristics were emphasized within the study.

After successful validation of the experimental model, the sloshing tests were performed with different fill volumes of 22%, 30%, and 70% at forced excitation frequencies (*f*_e_) of 0.8 to 1.2 times the first mode natural tank sloshing frequency in each case. Figure 2g,h are shown as a representative comparison between FlexiHMS and PZT sensors at 30% fill volume under resonant frequency *f*_e_ = *f*_1_ = 1.06171 Hz. The purpose here was to simply demonstrate how closely the two sensors tracked the same dynamic event. In Figure 2g, the steady phase time domain signals from both sensors showed clear periodic oscillations corresponding to the cyclic wall pressure variations generated during resonant sloshing. The overall waveform shape and oscillation frequency were consistent in both measurements, indicating that FlexiHMS and PZT captured the same fluctuations under identical excitation conditions. The corresponding FFT spectra in Figure 2h further confirmed this agreement, where both sensors successfully identified the dominant frequency at 0.998 Hz. The similarity of the identified spectral peak confirmed that FlexiHMS was capable of accurately detecting and resolving the fundamental dynamic frequencies of the system.

## 3 Results and Discussions

### 3.1 Impact of Liquid Fill Volume

In this section, the dynamic responses of FlexiHMS and PZT to slosh-induced pressures are examined for three liquid fill volumes. Forced excitation frequencies (*f*_e_) ranging from 0.8 *f*_1_ to 1.2 *f*_1_ were selected to cover resonant and nonresonant sloshing regimes, as mentioned earlier. For each combination of the excitation frequency and fill volume, three repeated trials were conducted, and the resulting peak pressure values were pooled for statistical analysis. It should be noted that FlexiHMS measured pressure, whereas the PZT sensor reflected structural vibration, thus, comparisons are limited to qualitative trends.

The degree of submersion of the sensors varied with liquid fill volumes. At 22% fill volume, the sensors were located just above the liquid-free surface, with approximately 1 mm submerged. At 30% fill volume, the sensors were submerged by approximately 1.6 cm in water, and at 70% fill volume, both sensors were fully submerged with 4.2 cm of water above FlexiHMS and 6.2 cm above PZT.

Figure 3 shows boxplots of peak pressure (FlexiHMS) and normalized peak voltage (PZT) readings extracted over several excitation cycles at various liquid fill volumes and forced excitations. Since sloshing-induced pressures naturally vary from cycle to cycle due to variation in wave elevations and wall impact intensities, box plots were utilized to statistically represent the distribution of peak values and to eliminate the influence of random fluctuations on quantitative analysis. Each column in the box plot depicts a set of peak values that were extracted from three repeated trials conducted under identical excitation conditions. To further highlight the repeatability of the measurements under identical excitation conditions, the averaged response obtained from the three repeated trials was also included as an additional data set in the box plots. For each box, the lower and upper edges correspond to the first and third quartiles, defining the interquartile range (IQR) of the measured peak. The whiskers extend to values that are within 1.5 times the IQR. Data points falling out of these bounds are isolated outliers, which are shown both above and below the whiskers. These isolated points correspond to occasional strong or weak-pressure events.

**Figure 3:**
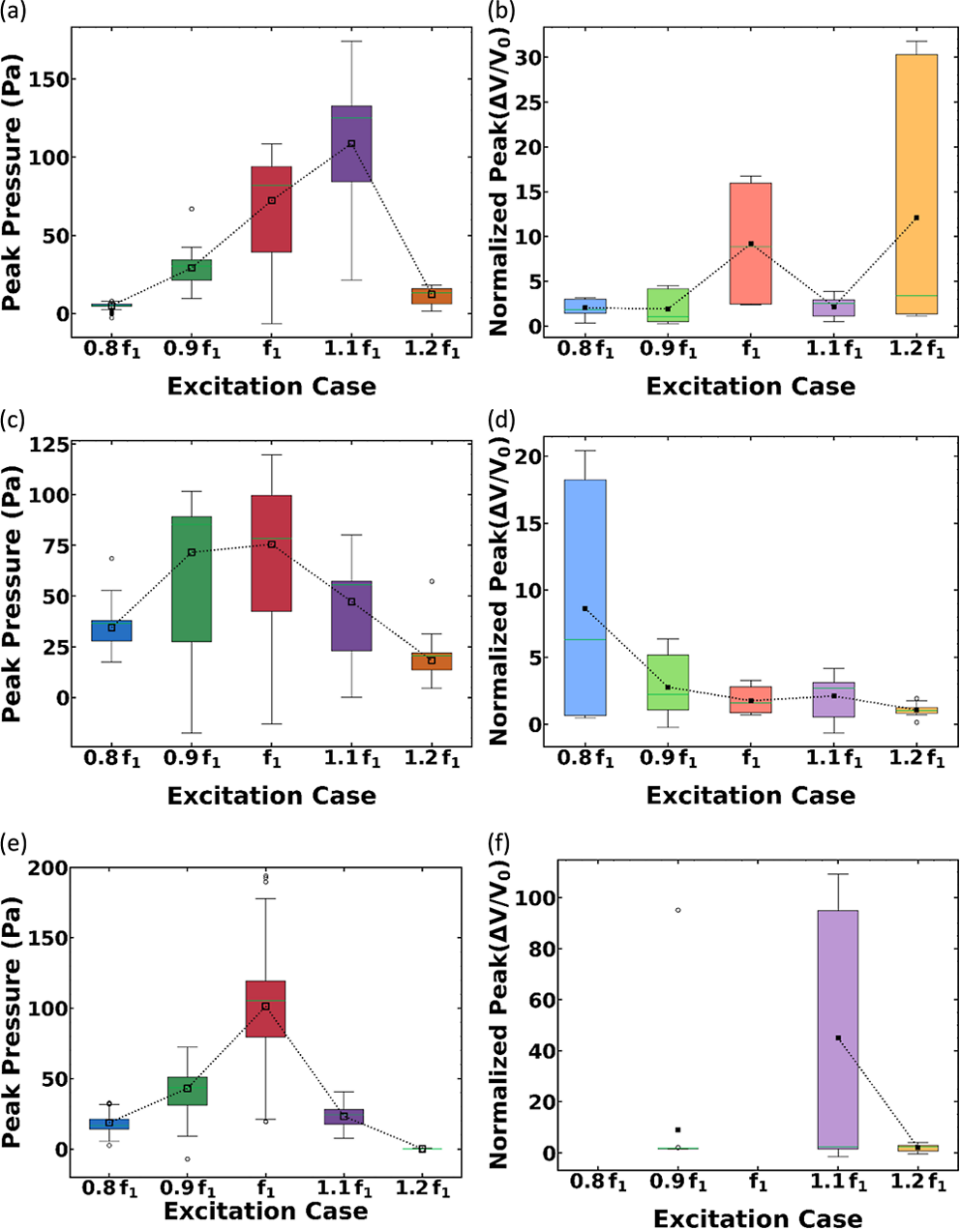
Statistical distribution of peak sloshing response under varying excitation frequencies. (a) Peak pressure measured using FlexiHMS at 22% fill volume, (b) corresponding normalized PZT response at 22% fill volume, (c) peak pressure measured using FlexiHMS at 30% fill volume, (d) corresponding normalized PZT response at 30% fill volume, (e) peak pressure measured using FlexiHMS at 70% fill volume, (f) corresponding normalized PZT response at 70% fill volume.

Figure 3a depicts the pressure responses of FlexiHMS at forced excitation frequencies of 0.8*f*_1_, 0.9*f*_1_, *f*_1_, 1.1*f*_1_, and 1.2*f*_1_ for 22% fill volume. At off-resonant excitation frequencies 0.8*f*_1_, 0.9*f*_1_, and 1.2*f*_1_, the mean peak pressures were low, with values of 4.22, 28.71, and 12.25 Pa, respectively. The narrow IQR in these cases indicated that the pressure was weak, with a small amplitude of surface waves. A stronger pressure response was observed as the excitation reached resonance (*f*_1_), with a mean peak pressure of 71.84 Pa, which further rose to 103.52 Pa at 1.1*f*_1_. The observed shift of maximum pressure response to 1.1 *f*_1_ was followed by a rapid decrease at higher excitation frequencies.^1^ Similar shifts have been reported in shallow water sloshing and are often interpreted as Duffing-type nonlinear behavior, where the peak moves away from expected resonance.^1,24^ This can happen because of wave steepening and fluid inertia. The corresponding box plots at *f*_1_ and 1.1*f*_1_ showed wider IQRs and extended whiskers, which indicate larger variability in pressure amplitudes. This variability is consistent with nonlinear sloshing behavior due to transitions from standing waves to progressive waves and the formation of hydraulic jumps near resonance. Experimental studies have shown that for shallow liquid depths, excitation close to the natural sloshing frequency led to the formation of traveling waves, hydraulic jumps, and solitary waves that generate localized and impulsive pressure on the tank walls.^21,25^

Figure 3b depicts the voltage responses for the PZT under the same *f*_e_ (0.8*f*_1_ to 1.2*f*_1_) for 22% fill volume. PZT exhibited a maximum response near the resonant frequency, where the mean normalized peak voltage increased from 1.19 V at 0.8*f*_1_ to 11.77 V at *f*_1_, followed by a decrease to 6.43 V at 1.2*f*_1_. Although the PZT did not directly measure pressure, its voltage response broadly followed the overall trend of the pressure response from FlexiHMS. However, the highest peak for PZT occurred at *f*_1_, whereas the FlexiHMS response peaked at near 1.1*f*_1_. This difference arose not only from the fundamentally distinct sensing mechanisms of the two sensors but also from the nonlinear sloshing behavior under shallow fill conditions. At low fill volume, the sloshing transitions from linear standing waves to nonlinear regimes that are characterized by traveling waves, hydraulic jumps, and localized wall impacts.^21^ These phenomena produce highly nonsinusoidal pressure fields with intermittent, high-amplitude contact events concentrated near the tank wall. Therefore, the wall pressure becomes strongly amplitude-dependent, and the maximum wall pressure may occur at excitation frequencies slightly higher than the linear natural frequency. Such nonlinear frequency shifts and jump phenomena in shallow water sloshing have been widely reported in experimental studies.^25^ FlexiHMS, which directly measured the local wall contact pressure, was sensitive to these localized pressure peaks and, therefore, captured the shifted maximum response beyond *f*_1_. In contrast, the PZT responded primarily to structural acceleration, which represents the global motion of the fluid-structure system and is insensitive to static pressure.^26^ Therefore, the PZT response naturally peaked near *f*_1_, while the FlexiHMS response reflects the nonlinear amplification of localized wall impacts at slightly higher excitation frequencies. The strong amplification observed near resonance in both sensors is consistent with classical sloshing behavior, where high-energy wave impacts near resonance lead to increased wave amplitudes.^7^

Figure 3c shows the results for 30% fill volume where FlexiHMS was partially submerged. At this fill volume, pressure loading arises due to free surface motion as well as continuous liquid contact leading to comparatively higher-pressure responses even at off-resonant frequencies as compared to the 22% fill volume. The FlexiHMS detected mean peak pressures of 34.36 Pa at 0.8*f*_1_, 71.76 Pa at 0.9*f*_1_, 75.54 Pa at *f*_1_, 47.22 Pa at 1.1*f*_1_, and 18.18 Pa at 1.2*f*_1_. The relatively small change in mean peak pressure between 0.9*f*_1_ and *f*_1_ indicated that the resonance effect was less pronounced as compared to 22% fill volume. The reduced peak response for this fill volume agreed with experimental findings that show that an increase in liquid depth enhanced inertial effects due to larger fluid mass in motion, which broadened the frequency response over a wider frequency range.^21^ Although the resonance peak for 30% fill volume was less pronounced, the box plots showed outliers and broader IQRs across multiple excitation frequencies, indicating higher variability in peak pressure responses. Experimental studies at similar fill volumes have reported that impact pressures depend more on local flow velocity and wave kinematics than on global sloshing amplitude, resulting in consistent cycle-to-cycle variability even under fixed excitation conditions.^27^

The partially submerged PZT response (Figure 3d) at 30% fill volume completely differs from the FlexiHMS trend. The highest mean normalized peak voltage occurred at 0.8*f*_1_ (10.13 V), followed by a continuous decrease to 4.69 V at 0.9*f*_1_, 2.6 V at *f*_1_, 1.75 V at 1.1*f*_1_, and 1.04 V at 1.2*f*_1_. This unusual behavior in response indicates that partial submergence of the sensor did not respond to the peak response solely. The PZT exhibited a different response compared with FlexiHMS, although the exact contribution of wall vibration or fluid loading cannot be separated.

Figure 3e depicts the FlexiHMS responses for 70% fill volume where the sensor was completely submerged. At this fill volume, the free-surface motion is strongly constrained and significantly damped by liquid inertia. The mean peak pressure response of FlexiHMS was moderate at off-resonant conditions with 18.77 Pa at 0.8*f*_1_ and 43.02 Pa at 0.9*f*_1_ but increased sharply to 101.45 Pa at *f*_1_. This rapid increment indicated that resonance could still excite internal sloshing modes even at high fill volumes. The box plot at *f*_1_ showed the largest IQR and whiskers, indicating strong response variability. Beyond resonance, the mean peak pressure dropped rapidly to 23.3 Pa at 1.1*f*_1_ and almost became negligible at 1.2*f*_1_ with a value of 0.26 Pa. The rapid pressure drop demonstrated the effective suppression of coherent sloshing at higher frequencies. However, despite reduced mean pressure, the presence of an outlier indicated persistent localized pressure fluctuations. This behavior was found consistent with a previous study by Lugni et al. where localized flow-focusing and near-wall jet formation could occur even when the overall free-surface motion was weak.^7^

The completely submerged PZT response (Figure 3f) at a 70% fill volume exhibited a distinctly different pattern. It was seen that no reliable mean values were obtained at 0.8*f*_1_ and *f*_1_. This behavior was further examined by time-domain analysis of the signals in Section 3.3. It was observed that the PZT sensor could not record any fluctuation signals at 0.8*f*_1_ and *f*_1_, resulting in a flat signal. Only a pronounced response was observed at 1.1*f*_1_, with a normalized mean peak voltage of 45.03 V accompanied by an exceptionally wide IQR and long whiskers. This suggested that under fully submerged conditions, the PZT sensor response was by localized wall vibration or internal flow-induced excitation rather than by global sloshing resulting in inconsistent response to sloshing-induced fluctuations. The intertrial repeatability of the FlexiHMS sensor showed a low coefficient of variation (below or nearly 10%) across the three trials for each fill volume and excitation frequency case (refer to Supporting Information, Appendix IV).

The variation in peak pressure across different fill volumes did not follow a monotonic trend but depended on both the fill level and excitation frequency. The 30% fill volume exhibited relatively higher pressure responses under several off-resonant excitation conditions (0.8*f*_1_, 0.9*f*_1_, and 1.2*f*_1_), whereas the 70% fill volume showed the highest response near the resonant condition (*f*_1_). At higher fill volume, the larger fluid mass and increased liquid depth likely contributed to stronger wall loading near the resonance. In contrast, at the super-resonant condition (1.1*f*_1_), the 22% fill volume exhibited the highest pressure response. Similar behavior has been reported in shallow-liquid sloshing systems, where nonlinear effects such as wave steepening, hydraulic jumps, and wave breaking can shift the maximum response away from the theoretical resonant frequency.^25,28^ These observations suggest that the measured pressure response is influenced by the combined effects of the liquid depth, resonance behavior, fluid inertia, and nonlinear sloshing dynamics, resulting in a condition-dependent variation across fill volumes.

### 3.2 Impact of Excitation Frequency

The effect of excitation frequency on sloshing dynamics was examined via time domain response evaluation as it helps analyze the evolution from the transient to steady phase regime. As the liquid is excited by forced excitations, it evolves through a transient phase where the sloshing peak amplitude fluctuates before reaching a steady phase where the response becomes repeatable.^7^ Two time windows were selected from each signal: 30–40s representing the transient phase and 340–350s representing the steady phase. Although time-domain signals were recorded for all of the excitation frequencies, only the responses corresponding to the resonant frequency *f*_1_ are presented in Figure 4 for all fill volumes, for representation.

**Figure 4:**
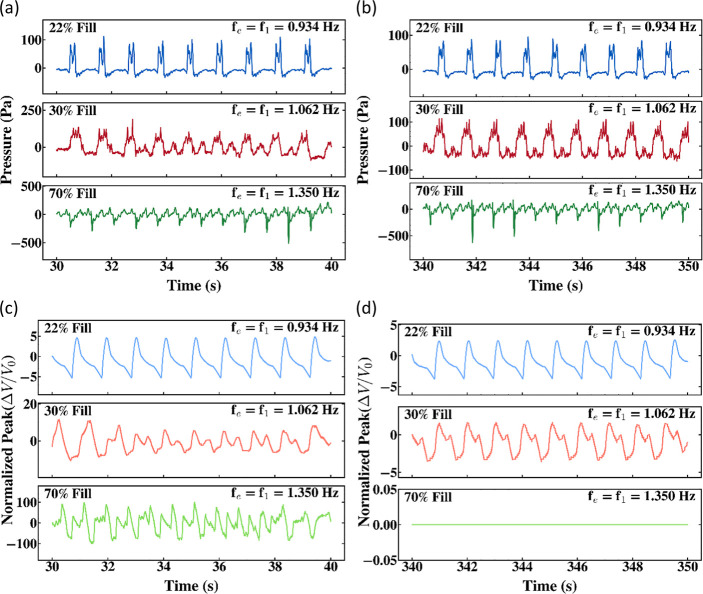
Time domain response for sloshing at resonant excitation frequency *f*_*e*_ = *f*_1_. FlexiHMS pressure signals at 22%, 30%, 70% fill volumes during (a) the transient phase (30–40s) showing progressive growth in oscillation and (b) the steady phase (340–350s) where the fluctuations reached stable and repeatable waveform; normalized PZT response during the (c) transient phase (30–40s) and (d) steady phase (340–350s) for the same excitation and fill volumes.

Figure 4a,b show the time domain responses for FlexiHMS at transient and steady phase, respectively. It was observed that the time-domain responses had multiple peak phenomena, which indicated multiple impacts on the tank wall after the sloshing wave was broken.^1^ At 22% fill volume, the pressure signals exhibited sharp intermittent pressure peaks, initially indicating an impulsive pressure loading caused by wave impacts on the wall. In the steady phase, the overall response variability minimized, but pronounced peaks with repeatable trend persisted, indicating sustained nonlinear sloshing. At this fill volume, oscillations were characterized by stronger peaks due to the strong free surface motions and wave breaking, typical of shallow water sloshing. At 30% fill volume, the transient phase showed higher pressure amplitudes with moderately irregular peaks, while the steady phase response became smoother and more repeatable with almost consistent peak magnitudes. As compared to the shallow fill case, the sloshing responses in this fill volume were noticeably more stable and repeatable across all the excitation frequencies, indicating a transition to a less impulsive flow regime. At 70% fill volume, both transient and steady phases showed similar pressure signal trends, but at the steady phase, the peak amplitudes became consistent with the consecutive peaks.

Figure 4c,d depict the transient and steady phase response of the PZT sensor, respectively, for all fill volumes. At 22% fill volume, the transient PZT signal exhibited a higher peak amplitude, which reduced and stabilized as the system reached a steady phase. At 30% fill volume, the transient phase clearly showed irregular voltage spikes that evolved into a more periodic and repeatable waveform during the steady phase, indicating the stabilization of wall impacts. At 70% fill volume, the transient phase showed irregular wave signals but the steady phase lacked a sustained periodic pattern. This suggested that under high fill conditions, the increased liquid inertia and viscous damping resulted in the PZT sensor being incapable of capturing any reliable signal.

Figure 5 presents the frequency spectra of the steady phases (340–380s) obtained from the FlexiHMS and PZT sensors for all liquid fill volumes and excitation frequencies. Figure 5a,b show the frequency spectra for sensors at 22% fill volume. In Figure 5a, FlexiHMS exhibited multiple prominent peaks across the frequency range. For excitation at 0.8*f*_1_ = 0.747 Hz, a strong peak appeared at 0.69 Hz, accompanied by higher harmonics that were equal to the multiples of the first prominent frequency. Similar frequency spectra were obtained for higher frequencies of 0.9*f*_1_ to 1.2*f*_1_. Compared to higher fill levels, the FFT spectra at 22% fill are relatively broader and exhibit a stronger harmonic content. This behavior is attributed to the nonlinear nature of shallow liquid sloshing. At shallow fill, sloshing transitions from linear standing waves to strongly nonlinear regimes characterized by wave breaking and hydraulic jumps.^1^ These processes introduce strong waveform distortion at the free surface and generate localized intermittent pressure impulses at the tank wall. Therefore, the wall pressure deviates from a sinusoidal form and contains higher-frequency components. In the frequency domain, this is reflected as the presence of higher harmonics and spectral broadening. Similar higher harmonic rich responses and nonlinear spectral features under shallow sloshing conditions have been experimentally reported in the literature.^25^ This behavior is further supported by corresponding power spectra (refer to Supporting Information, Appendix V), where enhanced harmonic energy at the shallow fill case is observed indicating higher wave energy and stronger nonlinear sloshing.

**Figure 5:**
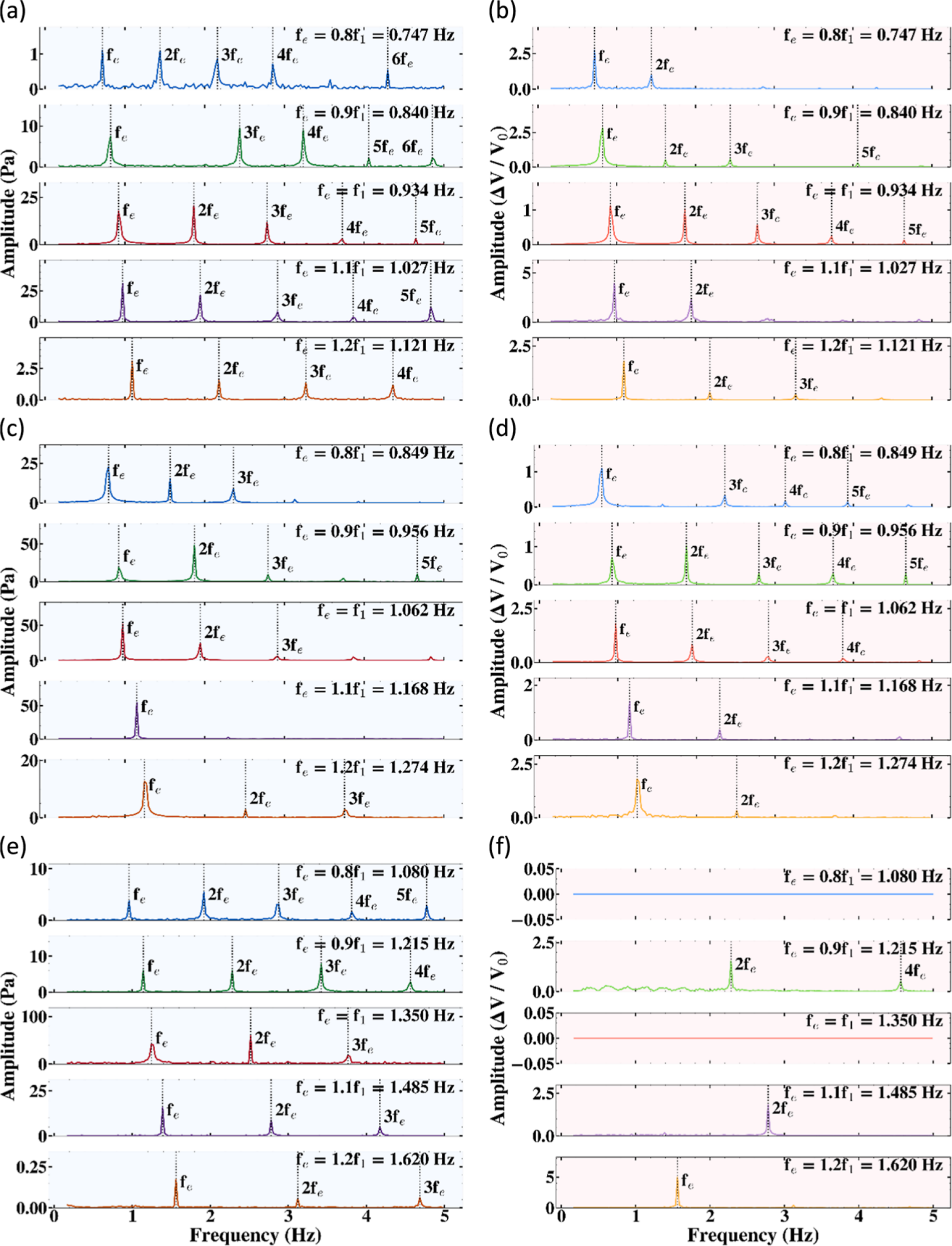
Spectral characteristics of sloshing induced response of FlexiHMS and PZT sensors at different fill volumes and excitation frequencies. (a) FlexiHMS FFT spectra at 22% fill volume for all excitation frequencies, where *f*_1_ = 0.934 Hz, (b) corresponding FFT spectra for PZT at 22% fill volume, (c) FlexiHMS FFT spectra at 30% fill volume for all excitation frequencies, where *f*_1_ = 1.062 Hz, (d) corresponding FFT spectra for PZT at 30% fill volume, (e) FlexiHMS FFT spectra at 30% fill volume for all excitation frequencies, where *f*_1_ = 1.35 Hz, (f) corresponding FFT spectra for PZT at 70% fill volume. Vertical dashed lines indicate the excitation frequency *f*_*e*_ for each condition followed by its higher harmonics.

In contrast, the PZT sensor spectra in Figure 5b show dominant peaks at the same frequencies for all fill conditions but weaker higher harmonics. This type of behavior has been observed in previous studies where partial decoupling between pressure harmonics and structural response has been reported in sloshing experiments, where impulsive loads do not always translate into proportional wall vibration.^7,27^

Figure 5c,d exhibit frequency spectra for FlexiHMS and PZT at 30% fill volume, respectively. The first peak identified in both sensors was similar, however, the amplitude of higher harmonics significantly reduced as we move further in frequency value. Near resonance and super resonance, it was observed that the spectral energy was primarily concentrated at the fundamental frequency, suggesting reduced wave breaking and weaker impulsive loading (refer to Supporting Information, Appendix V). This behavior corresponds to a critical depth regime where the resonance curve becomes more symmetric and the system response shows weak nonlinearity.^1^

Figure 5e,f show spectral response at 70% fill volume for FlexiHMS and PZT, respectively. FlexiHMS showed stronger energy toward higher frequencies, while fundamental components near excitation frequencies were comparatively weaker for 0.8*f*_1_ and 0.9*f*_1_. This indicated that the free surface sloshing was damped, and the pressure signal was influenced by internal flow motion and localized near-wall dynamics rather than coherent standing waves (refer to Supporting Information, Appendix V). Such behavior has been well documented in deep water sloshing studies, where increased liquid inertia due to higher volume leads to viscous dissipation.^21^ The PZT spectra showed a very different response with a flat frequency response at 0.8*f*_1_ and *f*_1_. However, at 0.9*f*_1_, 1.1*f*_1_, and 1.2*f*_1_, sharp isolated peaks appeared with higher frequencies. This type of response is known as hard-spring response where system responses are dominated by inertial effects rather than large-amplitude free surface motion.^1^

Further, it was observed from FFT spectra for all the fill volume cases, as the excitation frequency increased, the FFT peaks shifted toward the right in the plot to the value closer to the increased excitation frequency values. This further validated that the sensors successfully identified the imposed frequencies.

### 3.3 Comparison of FlexiHMS and PZT

The dynamic response of FlexiHMS was qualitatively evaluated against that of a commercially available PZT sensor under identical sloshing conditions. Since the two sensors operated via different transduction mechanisms, their outputs were independently normalized using their respective peak dynamic values to enable qualitative comparison of the dynamic response in terms of waveform shapes.

In the FlexiHMS sensor, the hierarchical microstructures introduce multiscale deformable features and air voids within the sensing film enabling progressive contact area evolution under continuously varying pressure conditions, thereby enhancing sensitivity.^24^ Compared to flat or simple microstructured surfaces, these structures exhibit low shape factor (defined as the ratio of compressed to uncompressed area) that promotes localized deformation and efficient stress concentration. This enables the sensor to detect low pressure variations (as low as ∼0.3 Pa) and respond to rapidly changing pressure fluctuations effectively, thereby resulting in faster response and recovery times.^25^ The presence of distributed sharp contact points facilitates rapid formation and disruption of conductive pathways that improve the sensor’s response. The FlexiHMS sensor had demonstrated a sensitivity improvement of approximately 316% and a reduction in response time by approximately 18% as compared to conventional hemispherical structures.^8^ These attributes make the FlexiHMS sensor suitable for capturing transient pressure variations including those occurring in liquid sloshing.

To better contextualize the performance of FlexiHMS, a comparative analysis with recently reported flexible piezoresistive pressure sensors is presented in Table 3 (adapted and updated from our previous work^8^). The comparison includes key parameters such as sensitivity, sensing range, response time, and sensor dimensions. The results show that FlexiHMS provides comparatively high sensitivity while maintaining a relatively wide sensing range. Although the response time is higher than some miniaturized sensors reported in literature, FlexiHMS has a significantly larger sensing area (40 × 40 mm^2^), which was intentionally designed for distributed pressure monitoring over larger surface regions relevant to sloshing applications.

**Table 3:** Performance Comparison of FlexiHMS Sensor with Recently Reported Flexible Piezoresistive Pressure Sensors

microstructure types	materials	maximum sensitivity (/kPa)	linear sensing range (kPa)	response time (ms)	sensor size (mm x mm)	refs
sandpaper microstructures	PDMS/rGO/CNT	0.341	0–1.6	126	10 × 10	^29^
Hump-like microstructure	rGO/FPU	9.448	0–9.2	40	20 × 8	^11^
		0.480	9.2–84.1			
		0.026	84.1–300			
multi-stage microdome	MXene/PDMS	5.57	0–111	82.62	5 × 5	^30^
		11.57	111–2500			
3D porous sponge	CNT/CB/TPU/PU	0.10	0–8	119	20 × 15	^31^
		0.01	8–23.3			
nano/micro-structures	MXene/dust-free paper	16.7	0–20	50	12 × 12	^32^
		10.6	20–40			
		6.1	40–100			
	MWCTs/Hemp fabric	0.2358	0–1	125	30 × 15	^33^
		0.02561	1–6			
		0.00307	6–30			
		0.00054	30–300			
hemisphere microstructure (FlexiHMS)	MWCNT/PDMS	25.33	0–60	360	40 × 40	this work^8^
		8.17	60–200			

Figure 6 depicts the steady phase responses of both sensors at 70% fill volume under varying excitation frequencies from 0.8*f*_1_ to 1.2*f*_1_. This case is presented as a representative high-fill condition to compare how the two sensors behave when wall–fluid coupling becomes weak. The responses of FlexiHMS and PZT were noticeably different in this regime. As seen in Figure 6a, FlexiHMS captured measurable pressure fluctuations across all of the excitation frequency range. Even at off-resonant frequencies (0.8*f*_1_ and 0.9*f*_1_), where the oscillations were irregular, small-amplitude pressure variations were clearly recorded. At *f*_1_, the pressure signal became slightly more structured, and occasional sharp pressure drops were visible. However, the pressure signals observed were not as periodic as those observed for 22% and 30% fill volumes (refer to Supporting Information, Appendix VI). At *f*_1_ and 1.1*f*_1_, the signal became more structured, with occasional sharp pressure drops that likely corresponded to localized wall interactions. Although the response weakened again at 1.2*f*_1_, the sensor continued to detect the dynamic pressure changes. This is typical of high fill volume sloshing, where the free surface motion is restricted and the response is dominated more by internal fluid movement than by large surface waves. Thus, it is important to note that the FlexiHMS sensor remained responsive, even when the pressure amplitudes were small.

**Figure 6:**
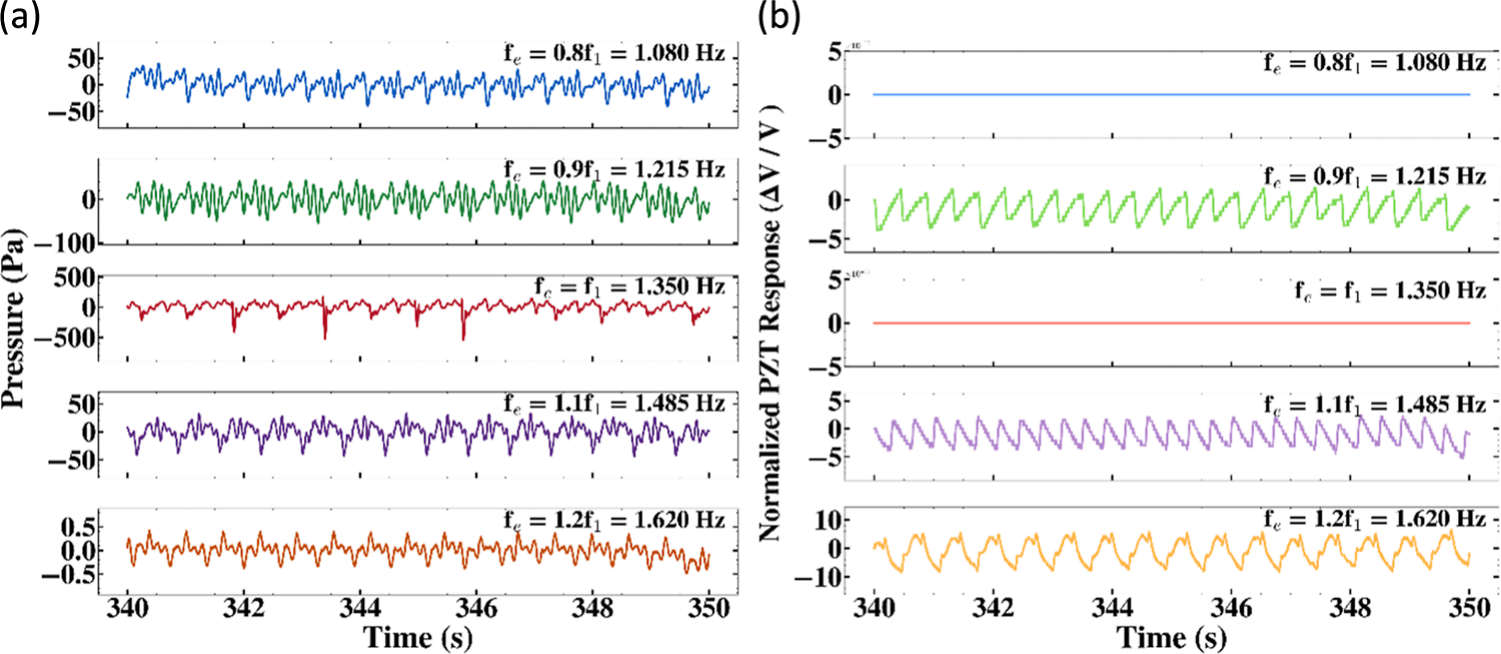
Representative steady state time-domain sloshing responses at 70% fill volumes under varying excitation frequencies; (a) FlexiHMS pressure signals at 0.8*f*_1_ to 1.2*f*_1_ showing localized pressure fluctuations and increased periodicity near resonance, (b) corresponding normalized PZT response under identical conditions with immeasurable responses at 0.8*f*_1_ and *f*_1_.

In contrast, the PZT response (Figure 6b) was negligible at 0.8*f*_1_ and *f*_1_, despite the presence of dynamic fluctuations, as detected by FlexiHMS (Figure 6a). A measurable response was observed at 0.9*f*_1_, *f*_1_, and 1.2*f*_1_, which indicated that PZT became active only when structural vibration was sufficiently amplified. This difference in behavior reflected the inherent limitation of piezoelectric sensing, which responded primarily to wall strain rather than localized pressure fluctuations. At high fill volumes, the larger fluid mass confined the free-surface motion and reduced the localized pressure fluctuations translating into structural deformation, i.e., the pressure field and wall vibration became partially decoupled.^5^

Under these conditions, FlexiHMS continuously detected even small-scale dynamic pressure signals, whereas PZT responded only when the structural vibration became sufficiently pronounced. The time domain analysis of 70% fill volume helped interpret the discrepancy observed in the statistical analysis in Figure 3f, highlighting the differences between direct wall pressure sensing and structural strain-based sensing in high-fill sloshing regimes.

The comparison analysis in this section helps answer the key questions raised at the beginning of this study. First, the results clearly showed the capability of FlexiHMS in capturing dynamic sloshing-induced pressures under continuous oscillatory motions. It recorded stable time-domain signals and consistent statistical trends across all fill volumes and excitation frequencies under repeated trials. This confirmed the reliability of the FlexiHMS for dynamic pressure monitoring. The presence of hierarchical microstructures enhanced its compressibility by creating voids and sharp contact points. These structural modifications enabled efficient deformation of the sensor even under very small pressure variations, allowing it to capture subtle dynamic fluctuations. Second, it was noticed that both liquid fill volume and excitation frequency strongly influenced the magnitude and variability of the pressure response. At near-resonance, pressure amplification and peak pressure dispersion were observed. Finally, comparison with PZT revealed that FlexiHMS continued to exhibit measurable pressure fluctuations at higher fill volumes, whereas the PZT response diminished under certain conditions. This indicated that direct wall pressure sensing provided a clearer representation of sloshing dynamics in regimes where structural deformation could not reflect fluid activity.

## 4 Conclusions and Future Work

This work demonstrated that flexible piezoresistive sensors can serve as a nonintrusive and conformal alternative to conventional rigid transducers for dynamic pressure monitoring in sloshing environments. The feasibility of a previously developed laser-engraved hierarchically microstructured flexible pressure sensor (FlexiHMS) was evaluated by measuring slosh-induced wall pressures in a laboratory-scale rectangular tank under a controlled harmonic excitation.

Sloshing experiments were conducted across subresonant, resonant, and super-resonant regimes (0.8*f*_1_ to 1.2*f*_1_) and at liquid fill volumes of 22%, 30%, and 70%. Free-decay tests validated the test protocol by confirming that the dominant excitation frequencies deviated by less than 5% from theoretical predictions. Under forced excitation, FlexiHMS reliably captured dynamic wall pressures over a wide range (0.3–127 Pa) and exhibited clear resonance-driven amplification behavior. Statistical analysis of steady-state responses revealed increased dispersion and intermittency near resonance, due to nonlinear effects such as wave steepening and localized impact events. Across all test conditions, the sensor showed stable waveforms and strong repeatability across the three independent trials.

A comparative assessment against a commercial PZT sensor highlighted fundamental differences arising from the sensing mechanisms. While the PZT response peaked near the first natural sloshing frequency due to its acceleration-sensitive nature, FlexiHMS exhibited maximum responses at slightly higher excitation frequencies at the lowest fill volume, reflecting pressure-dominant nonlinear interactions under shallow liquid conditions. At higher fill volumes, the PZT response diminished under certain conditions, whereas FlexiHMS continued to detect measurable pressure fluctuations. These differences arise from the distinct sensing principles of the two sensors, where FlexiHMS measured wall pressure, while the PZT primarily reflected structural vibration. Despite these differences, both sensors demonstrated a stable periodic response.

The ability of FlexiHMS to be directly adhered to tank walls without structural modification represents a key advantage over conventional rigid sensors, especially for curved, thin-walled, or flexible containers, and a strong potential for practical deployment in dynamic fluid-structure interaction systems.

A limitation of the present study is that experiments were performed in a single-tank geometry, with a single working fluid and fixed sensor positions. Moreover, no accelerometer-based wall measurements or numerical simulations were included in this study. The sensors were mounted using a thin silicone adhesive layer for conformal attachment. However, its influence on dynamic pressure transmission needs further investigation compared with rigid flush-mounted configurations.

Future work will extend to distributed sensor arrays, curved and flexible tank geometries, and more-complex excitation scenarios. Preliminary liquid oscillation tests showed that FlexiHMS provided a reliable amplitude-dependent response with a measurable phase lag. However, a more comprehensive dynamic calibration will be carried out in a future work. Furthermore, the effect of different fluid types and thermal conditions will be examined to assess robustness and cross-sensitivity effects in the sensor. In parallel, the study seeks to encourage collaborative development of open-access sloshing data sets. Such open resources would accelerate the cross-validation of sensor technologies, improve model development, and enable more reproducible research in sloshing.

## Supplementary Material





## Data Availability

The data set used in this work will be made available on request to the corresponding author.

## Notes

Liquid oscillation test for FlexiHMS dynamic response; thermal and curvature dependent sensor response; excitation frequency uncertainty analysis; FlexiHMS intertrial repeatability; power spectral analysis of FlexiHMS responses; and time-domain analysis of FlexiHMS and PZT sensor responses (PDF)
Sloshing.mp4 (MP4)
