# Supplementary material for: Nonintrusive Dynamic Pressure Monitoring of Sloshing Liquid Using a Hierarchically Microstructured Flexible Sensor
Source: Langmuir. 2026 Jun 18;42(27):19510–21. doi: 10.1021/acs.langmuir.6c01078 (PMC13374381; doi:10.1021/acs.langmuir.6c01078)
Supplement: Supplementary file 1 [file la6c01078_si_001.pdf]

SUPPORTING INFORMATION

Non-intrusive Dynamic Pressure Monitoring of Sloshing Liquid using a Hierarchically Microstructured Flexible Sensor

Parul Thapa[1], Shrutidhara Sarma[2*]
[1,2]Dept. of Mechanical Engineering, IIT Jodhpur, Jodhpur, Rajasthan 342030, India

Corresponding Author*:
Dr. Shrutidhara Sarma
Associate Professor,
Dept. of Mechanical Engineering,
IIT Jodhpur, Rajasthan-342037
Email: shrutidhara.sarma@tu-braunschweig.de

Number of pages: 8
Number of figures: 5
Number of tables: 1

Table of Contents

Appendix I: Liquid Oscillation Test for FlexiHMS' dynamic response........S2
Appendix II: Thermal and Curvature dependent sensor response........S3
Appendix III: Uncertainty in Excitation Frequency Estimation........S4
Appendix IV: Inter-Trial Repeatability of Experiments........S5
Appendix V: Power Spectral Analysis of FlexiHMS........S6
Appendix VI: Time-Domain Analysis of Sensor Response........S7

Appendix I: Liquid Oscillation Test for FlexiHMS' dynamic response

A preliminary liquid oscillation test was performed to evaluate the dynamic response of FlexiHMS under oscillatory liquid loading. The FlexiHMS sensor was attached at the bottom of a cylindrical tank partially filled with water. A circular disc plunger was moved vertically using a linear motorized stage with sinusoidal excitation at 1.5 Hz and displacement amplitudes of 2, 3, and 4mm. For each test, displacement input and corresponding sensor output were recorded and analyzed. The time-domain responses shown in Fig.S1 (a) indicate that the sensor output followed the imposed sinusoidal displacement input with measurable phase delay of 35.2°, 48.5°, and 31.3° for 2, 3, and 4 mm amplitudes, respectively. The corresponding dynamic gains were 0.071, 0.088, and 0.086 Pa/mm. Moreover, the normalized pressure response (Fig. S1 (b)) increased from 0.569 at 2mm to 0.717 at 3mm and 0.968 at 4mm with linear trend ($R^2 = 0.9786$). The results demonstrated that FlexiHMS can reliably track periodic liquid pressure variations under oscillatory excitations.

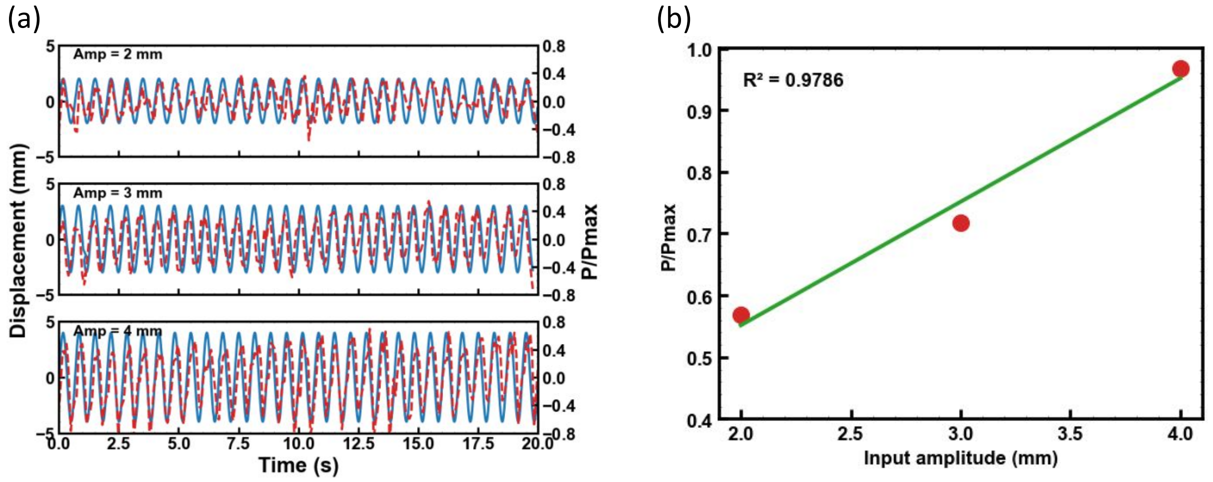

Fig. S1 *(a) Time-domain displacement input (blue) and normalized pressure response (P/P_{max}, red) for excitation amplitudes of 2, 3, and 4 mm at frequency 1.5 Hz. (b) Normalized pressure response (P/P_{max}) as a function of input displacement amplitude, showing linear trend ($R^2 = 0.9786$).*

Appendix II: Thermal and Curvature dependent sensor response

The FlexiHMS sensor maintained high linearity under different temperatures, with a mild increase in slope but minimal hysteresis, indicating a stable behaviour at varying temperatures (Fig. S2).

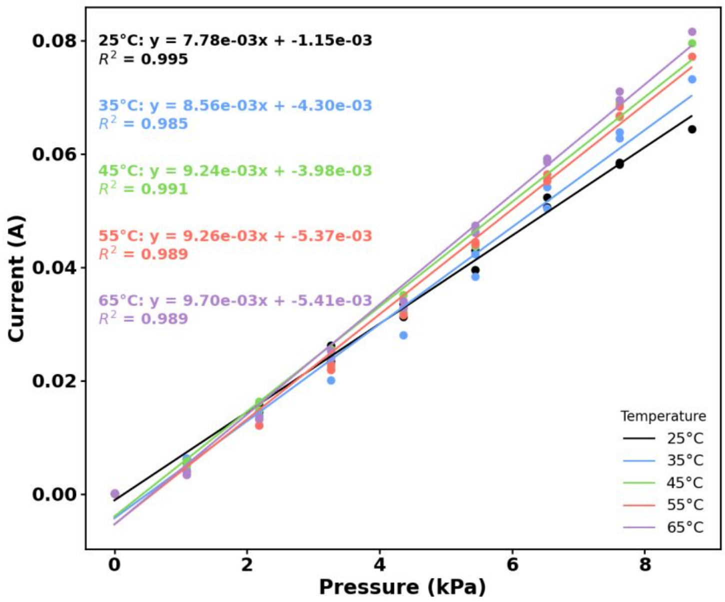

Fig. S2 *FlexiHMS sensor response at different temperatures (25-65 °C)*

The curvature dependent test results, Fig. S3, show that the FlexiHMS sensor exhibited ~16% reduction in response under curved (Radius=60mm) conditions but maintained stable and repeatable behaviour.

(a) (b)

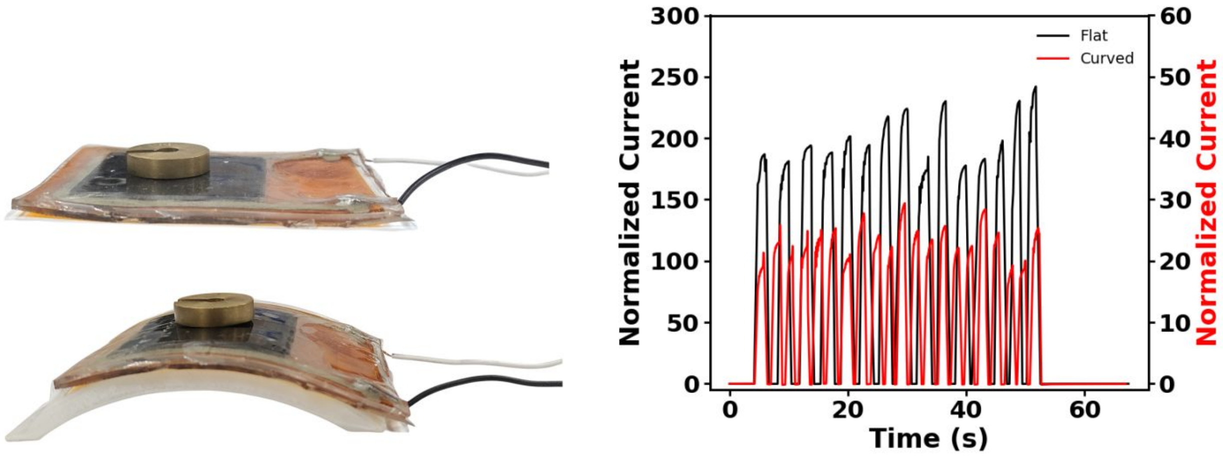

Fig. S3 *(a) Actual image of FlexiHMS loaded under flat and curved (R=60mm) conditions (b) corresponding response of FlexiHMS*

It is to be noted that for the curvature-dependent experiments the load was manually applied and removed in both cases. As a result, a slight difference in the time alignment of the signals and inconsistency in the maximum response were observed.

Appendix III: Uncertainty in Excitation Frequency Estimation

The effective sloshing frequency (f_e) was determined experimentally from high-speed (60 fps) video recordings by counting wave-wall impacts as-

$$f_e = \frac{N}{t} \tag{S1}$$

Considering there is no uncertainty in counting (N), the total uncertainty was estimated using standard error propagation as (*Holman, 2012*)-

$$\frac{\Delta f}{f} = \sqrt{\left(\frac{\Delta t}{t}\right)^2} \tag{S2}$$

where $\Delta t = \frac{1}{fps} = \frac{1}{60} = 0.0167$s (1 frame at 60 fps) corresponds to the temporal resolution of video acquisition.

Considering a 30 s window for analysis, the uncertainty comes out to be:

$$\frac{\Delta f}{f} = \sqrt{\left(\frac{0.0167}{30}\right)^2} = 5.6 \times 10^{-4} = 0.056\ \%$$

Thus, uncertainty was found as □± 0.056 % across all fill volumes.

Reference:

- Holman, J. P. (2012). J.P. Holman (8th ed.). McGraw-Hill series in mechanical engineering.

Appendix IV: Inter-Trial Repeatability of Experiments

The inter-trial variability of the FlexiHMS sensor was evaluated by computing the mean peak pressure, standard deviation (SD) and coefficient of variation (CV) across the three trials for each fill volume and excitation frequency cases, as listed in Table S1. The results indicated good repeatability across most excitation cases with relatively low CV values (below or nearly 10%). In some cases, the increase in variability was seen which can be contributed to increase in nonlinear sloshing dynamics.

Table S1. *Repeatability analysis of FlexiHMS sensor showing mean peak pressure (±SD) and coefficient of variation (CV) across three trials for different fill volumes and excitation cases.*

Fill volume	Excitation Case	Mean ± SD (Pa)	Coefficient of variation (CV)
22%	0.8f1	5.37 ± 0.47	8.71%
	0.9f1	32.39 ± 3.12	9.64%
	f1	86.70 ± 8.04	9.28%
	1.1f1	131.44 ± 7.19	5.47%
	1.2f1	14.35 ± 2.09	14.58%
30%	0.8f1	37.49 ± 0.70	1.86%
	0.9f1	86.89 ± 3.11	3.58%
	f1	91.46 ± 16.94	18.52%
	1.1f1	56.31± 1.50	2.67%
	1.2f1	21.28 ± 0.95	4.44%
70%	0.8f1	20.39 ± 7.04	34.54%
	0.9f1	47.64 ± 9.67	20.29%
	f1	108.04 ± 2.83	2.62%
	1.1f1	25.41 ± 3.58	14.08%
	1.2f1	0.31 ± 0.04	13.26%

Appendix V: Power Spectral Analysis of FlexiHMS

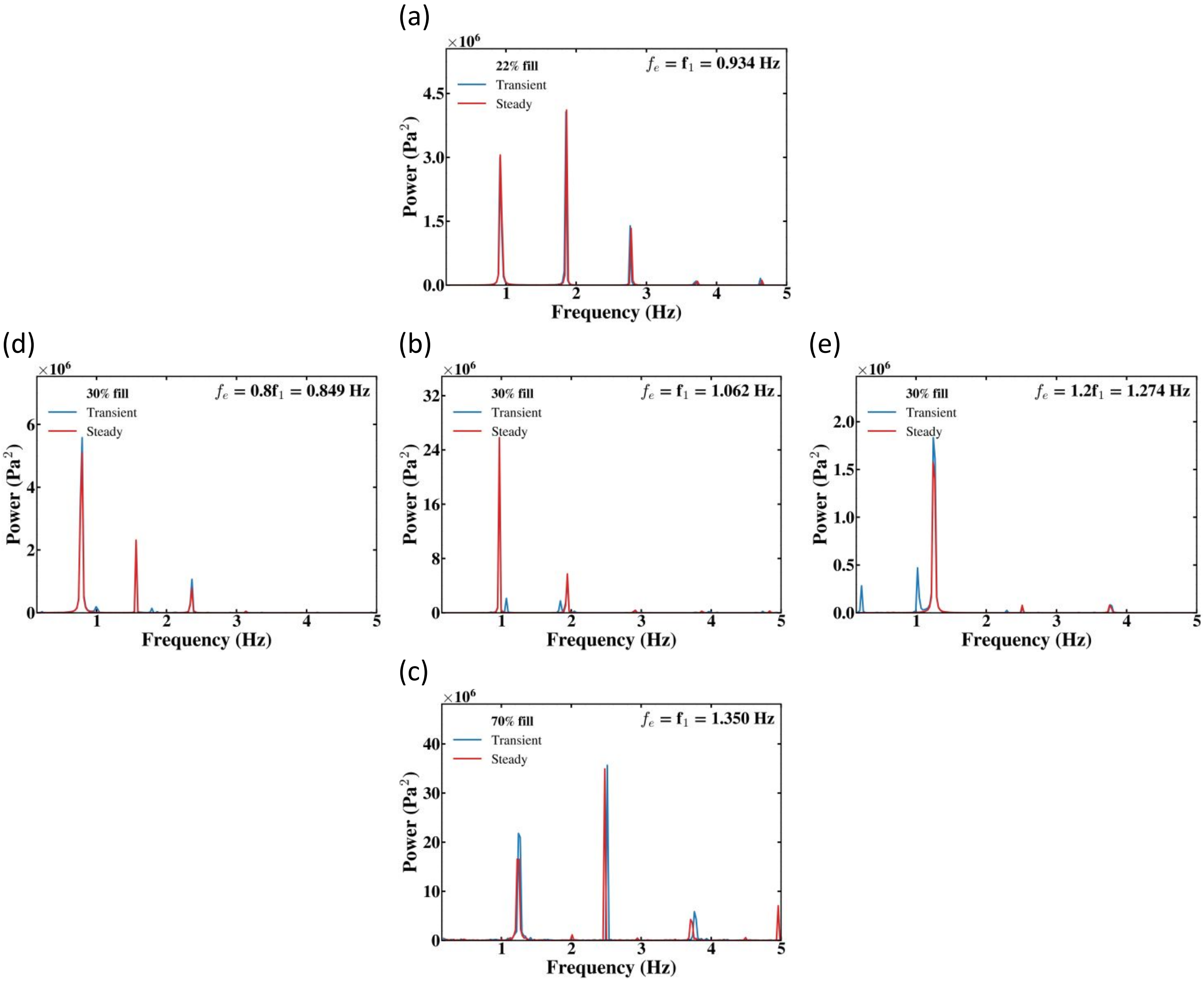

Fig. S4 *Power spectral analysis of FlexiHMS responses during transient and steady phases: (a) 22% fill volume for forced excitation at f_1 = 0.934 Hz, (b) 30% fill volume at f_1 = 1.062 Hz, (c) at 70% fill volume at f_1 = 1.35 Hz. 30% fill volume for excitation at (d) 0.8 f_1 and (e) 1.2 f_1. The spectra were obtained from the squared FFT magnitude of the pressure signal and overlaid for transient (30–70 s) and steady (340 - 380 s) phases to illustrate the evolution of spectral energy during sloshing.*

Fig. S4 depicts that at lower fill volume (22% fill), the power spectra exhibited broader energy distribution and the presence of stronger harmonics. As the fill volume increased (30% fill), the spectral energy became more concentrated around the fundamental frequency, with reduced harmonic content. For excitation frequencies above and below resonance at intermediate fill volume, spectral energy was concentrated near the fundamental frequency with reduced higher-order components, which indicated increased damping and reduced free-surface wave activity. However, with higher fill volume (70% fill), the second harmonic energy exceeded the fundamental frequency, indicating that nonlinear wave interactions remained important even at higher fill levels.

Appendix VI: Time-Domain Analysis of Sensor Response

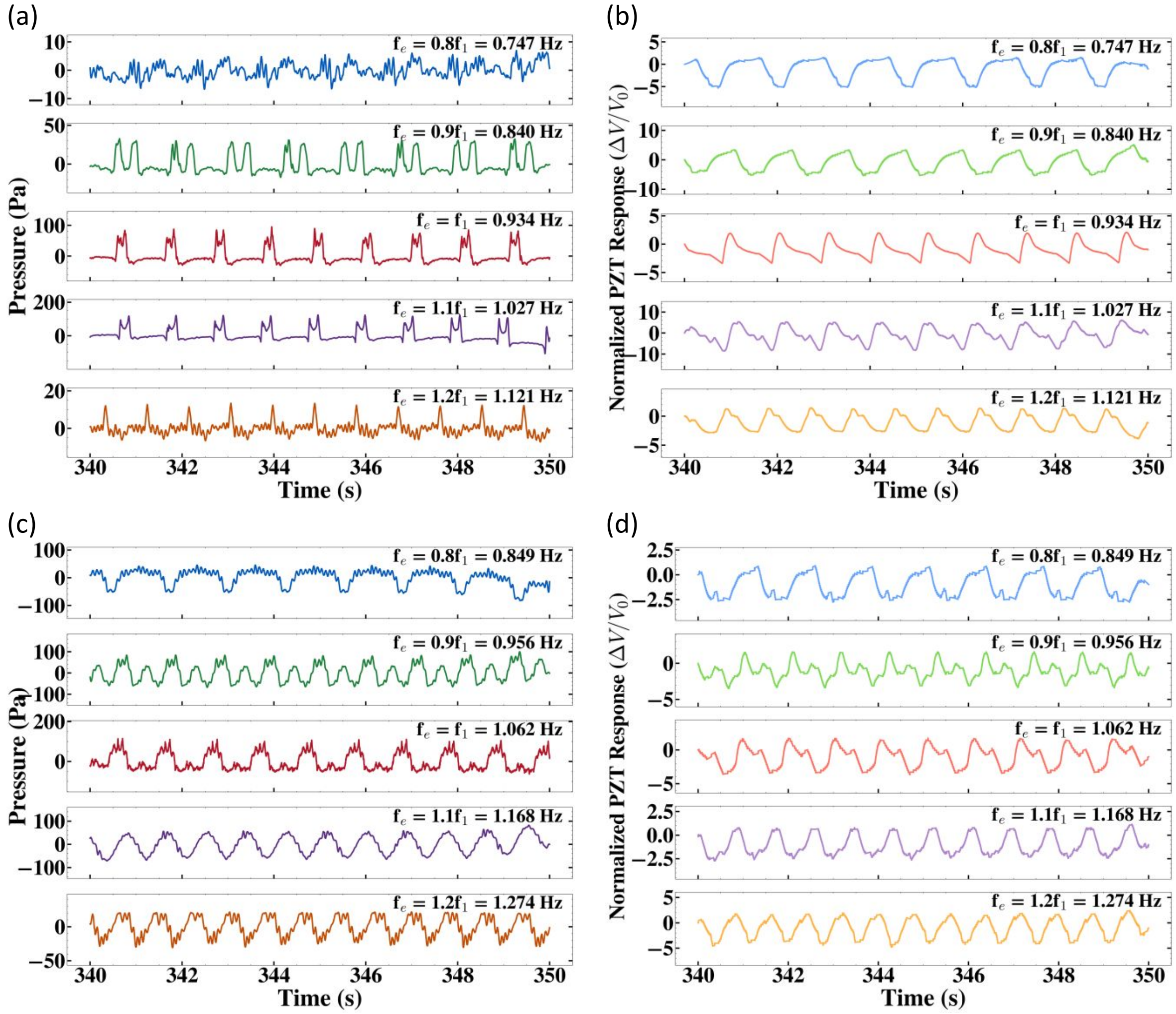

***Fig. S*5** *Time-domain analysis of responses from both FlexiHMS and PZT during steady phase. (a) FlexiHMS response at 22% fill volume for all excitation frequencies, where f_1=0.934 Hz, (b) Corresponding response for PZT at 22% fill volume, c) FlexiHMS response at 30% fill volume for all excitation frequencies, where f_1=1.062 Hz, (d) Corresponding response for PZT at 30% fill volume.*

In Fig. S5(a), at 22% fill volume, the FlexiHMS pressure response was weak and irregular with poor repeatability at $0.8f_1$ but became periodic as the excitation approached resonance. At $0.9f_1$ and f_1, the response peaks occurred in a more repeatable and phase-locked manner, indicating strong resonant sloshing. The occurrence of double peaks in the pressure signals indicated two impacts on the tank wall within a single excitation cycle. When the excitation increased beyond resonance f_1 and $1.2f_1$ the amplitude reduced and the waveform became less sharp.

In Fig S5(b), the PZT response at 22% fill followed a similar trend but the stronger sloshing responses were observedf_1 and 1.1 f_1. The spectra got narrower as the excitation frequency increased.

At 30% fill volume, Fig. S5(c), the FlexiHMS pressure signals showed broad, low-frequency oscillations at 0.8f_1. At 0.9f_1, the pressure signal became more structured and primary peak with a shoulder was observed in each excitation cycle. At f_1, the pressure peaks were fairly stronger with the secondary peaks becoming weaker and eventually diminishing.

The corresponding PZT responses in Fig. S5(d) exhibited wave trends similar to FlexiHMS, across all frequencies. At 30% fill volume, both sensors successfully captured the sloshing dynamics at different excitation frequencies as the motion became less violent and more organized compared to 22% fill.
